# Benchmarking multi-ancestry prostate cancer polygenic risk scores in a real-world cohort

**DOI:** 10.1371/journal.pcbi.1011990

**Published:** 2024-04-10

**Authors:** Yajas Shah, Scott Kulm, Jones T. Nauseef, Zhengming Chen, Olivier Elemento, Kevin H. Kensler, Ravi N. Sharaf

**Affiliations:** 1 Englander Institute for Precision Medicine, Weill Cornell Medicine, New York City, New York, United States of America; 2 Department of Physiology and Biophysics, Weill Cornell Medicine, New York City, New York, United States of America; 3 Department of Medicine—Hematology and Medical Oncology, Weill Cornell Medicine, New York City, New York, United States of America; 4 Department of Population Health Sciences, Weill Cornell Medicine, New York City, New York, United States of America; 5 Department of Medicine–Gastroenterology and Hepatology, Weill Cornell Medicine, New York City, New York, United States of America; University of Virginia, UNITED STATES

## Abstract

Prostate cancer is a heritable disease with ancestry-biased incidence and mortality. Polygenic risk scores (PRSs) offer promising advancements in predicting disease risk, including prostate cancer. While their accuracy continues to improve, research aimed at enhancing their effectiveness within African and Asian populations remains key for equitable use. Recent algorithmic developments for PRS derivation have resulted in improved pan-ancestral risk prediction for several diseases. In this study, we benchmark the predictive power of six widely used PRS derivation algorithms, including four of which adjust for ancestry, against prostate cancer cases and controls from the UK Biobank and All of Us cohorts. We find modest improvement in discriminatory ability when compared with a simple method that prioritizes variants, clumping, and published polygenic risk scores. Our findings underscore the importance of improving upon risk prediction algorithms and the sampling of diverse cohorts.

## Author summary

Prostate cancer incidence is disproportionally higher in men of African ancestry when compared to Caucasian counterparts. However, disease risk prediction in these individuals remains poor. Algorithmic development has yielded improved risk prediction in diverse populations for select diseases. We benchmark the most commonly used algorithms and find modest improvements for prostate cancer prediction in men of African ancestry.

## Introduction

Prostate cancer (PCa) is the most commonly diagnosed tumor type in American men, accounting for 26% of all cancer cases [1]. Several genetic and environmental factors such as age, body mass index (BMI), family history and ancestry are strong risk factors for developing PCa [2]. Men of African ancestry have 1.5-fold higher incidence and those of Asian ancestry have a decreased incidence rate when compared to non-Hispanic white men [3,4]. However, the genetic basis for ancestry-linked disparities remains unclear. PCa is a heritable cancer where 58% of disease risk has been linked to genetic factors [5]. It is hypothesized that genetic predisposition may contribute to ancestry-linked risk.

Although genome-wide associations studies (GWASs) have identified several single nucleotide polymorphisms (SNPs) associated with PCa risk, they have primarily been conducted in European-ancestry cohorts [6–8]. Polygenic risk scores (PRSs), which combine risk associated with multiple variants into a single risk score, are usually created using SNPs derived from these GWASs. Consequently, PRSs may share the population-bias of the European ancestry-based GWAS and cannot be confidently applied to non-European populations. These biases are associated with differences in linkage disequilibrium, causal variant status and differing causal variant allele frequencies across populations [9]. Admixture mapping and meta analyses have revealed rare and common risk alleles specific to men of African ancestry [10–12]. The challenge of integrating genetic findings with the well-powered European-based GWAS results has led to the development of several methods that correct the biases within the European ancestry results [13–17]. The variability in complexity and correction efficiency across these methods presents an opportunity to systematically analyze their performance in PCa.

PRS-stratified prostate-specific antigen (PSA) screening may help reduce overdiagnosis and subsequent over-treatment associated with PSA screening alone [18]. However, given ancestry-associated PCa incidence and outcome biases, it is imperative that risk scores work equally well across populations in order to limit disparities.

In this study, we first evaluated the performance of several published polygenic risk scores in the UK biobank cohort. Next, we trained polygenic risk score models using the largest GWAS summary data from the ELLIPSE Prostate Cancer Meta-Analysis cohorts and tuning dataset from the UK Biobank. Model performance was evaluated in held-out individuals from the UK biobank as well as in individuals from the held-out All of Us cohort.

## Results

### Population ancestral assignment

We sought to stratify the UK Biobank cohort by ancestry so as to evaluate the efficacy of multi-ancestry PRSs. Extensive quality control was performed prior to ancestry assignment. We began with the 488,377 individuals who had genotypic data. We removed 1,807 individuals who were considered outliers in heterozygosity, had putative sex chromosome aneuploidy, or had excess relatives according to the quality control procedures completed by the UK Biobank.

Continental global ancestry assignment was performed by integrating genotypic data and self-reported ethnicity, similar to previous work [19]. UK Biobank participants were split into three populations (African (AFR) males = 3,846, Asian (ASN) males = 6905. European (EUR) males = 209,894) by using their self-reported answer to the question “What is your ethnic group?”.

**Fig 1:**
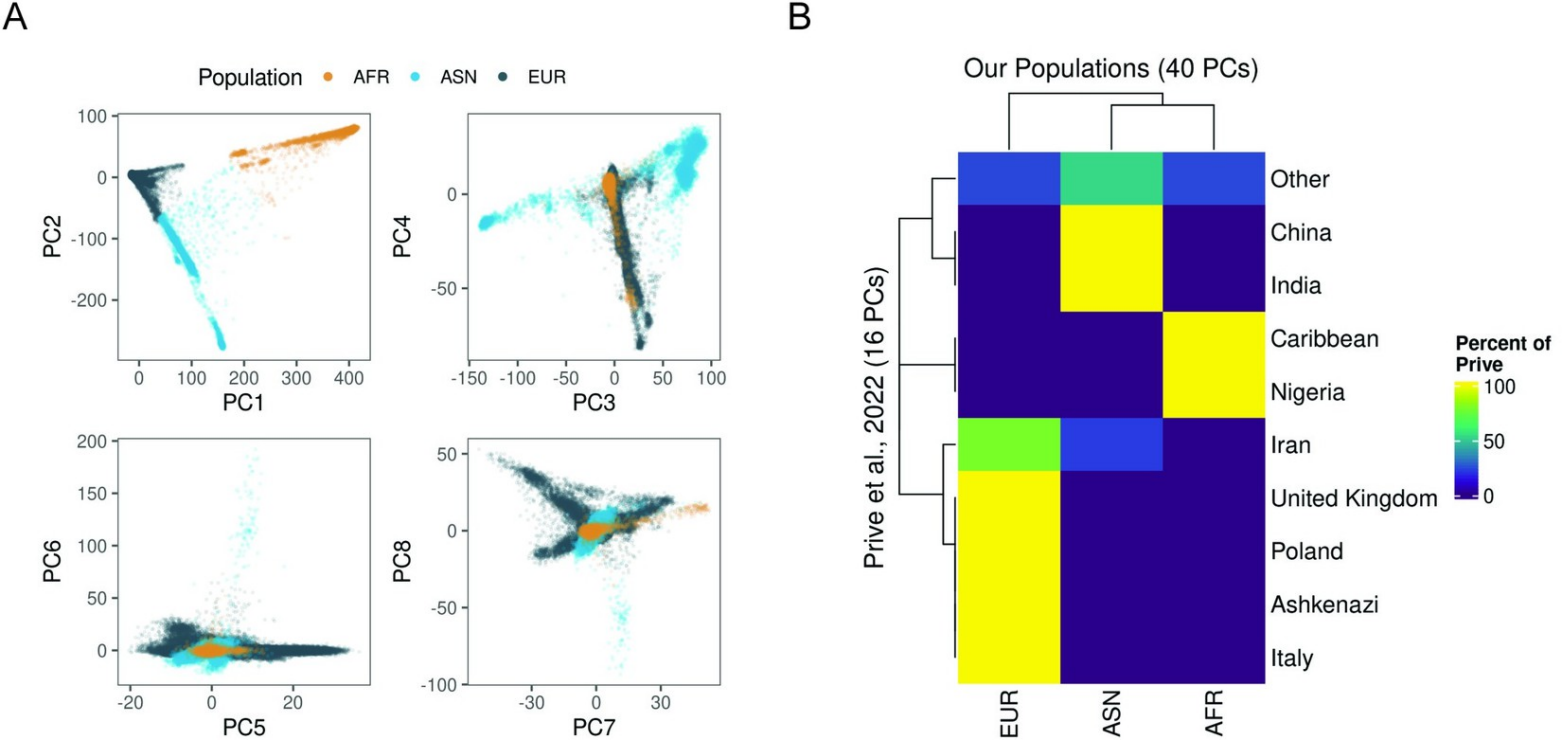
(A) Ancestry assignment of UK Biobank participants based on genetic principal components. (B) Comparison of ancestry assignment between those made within Prive et al., 2022 [19] and those made by us.

Next, we performed k-means clustering (k = 3) based on the Euclidian distance across the first 40 genetic principal components. The entire cohort was included in the clustering without regard for their questionnaire-derived population label. We integrated genetic and questionnaire data by assigning each cluster an ancestry based on the most prevalent self-reported population of the constituent individuals. This yielded high concordance between genetic and questionnaire data. 97% of all individuals in cluster 1 were considered ASN, 99% of all individuals in cluster 2 were AFR and 99% of all individuals in cluster 3 were EUR. Participants whose self-reported population disagreed with the computed population or who were in the 99^th^ percentile of distance from the cluster’s center were annotated as a population termed Other (n = 2,381, **Fig 1A**).

We further assessed population assignment by comparing our clusters with published country-level assignments based on the top 16 principal components. This revealed high concordance between annotations. Moreover, participants that did not fall into our 3-population buckets were identified as Other, Iranian, and Asheknazi by Privé et al., 2022 [19]. Interestingly, the use of additional principal components enabled population classification of samples annotated as Other by Privé et al., 2022 [19]. This led to the addition of 2,438, 4,882 and 2,406 samples to AFR, ASN and EUR groups, respectively (**Fig 1B**).

Finally, we limited our dataset to all males who were categorized into either AFR, ASN, or EUR. This resulted in a final dataset of 220,245 participants. Population assignments determined from the k-means clustering were utilized for all analyses.

### Assessment of published genetic risk scores

We annotated participants as cases and controls based on ICD-9, ICD-10, and self-reported information on prostate cancer status prior to conducting any genetic analyses. This process identified 13,097 cases and 207,148 controls in our dataset. Stark differences in population grouping, birth year, smoking, alcohol, and vital status were observed between cases and controls (**S1 Table**). Next, we aimed to ascertain the accuracy of published polygenic risk scores in our cohort. These scores were not derived from the UK Biobank and included both those which were ancestry-informed and those that were generated in a homogenous European population (**S2 Table**).

Fitting of polygenic risk scores involved a random stratified 2:1 split of our dataset into tuning and testing. The split was stratified by population grouping and case-control status. No differences in prostate cancer status or risk factors such as birth year, BMI, smoking status, or alcohol usage were identified (**S3 Table**). Publicly available polygenic risk scores were evaluated in the testing dataset. Briefly, we trained logistic models on scores obtained from allelic scoring of risk alleles and the first 10 genetic principal components using the entire testing dataset with cross validation, regardless of population grouping. Evaluation of predictive power in the testing cohort was performed in two ways, both, evaluation within the entire cohort and evaluation within each annotated population (**S1A Fig**).

**Fig 2:**
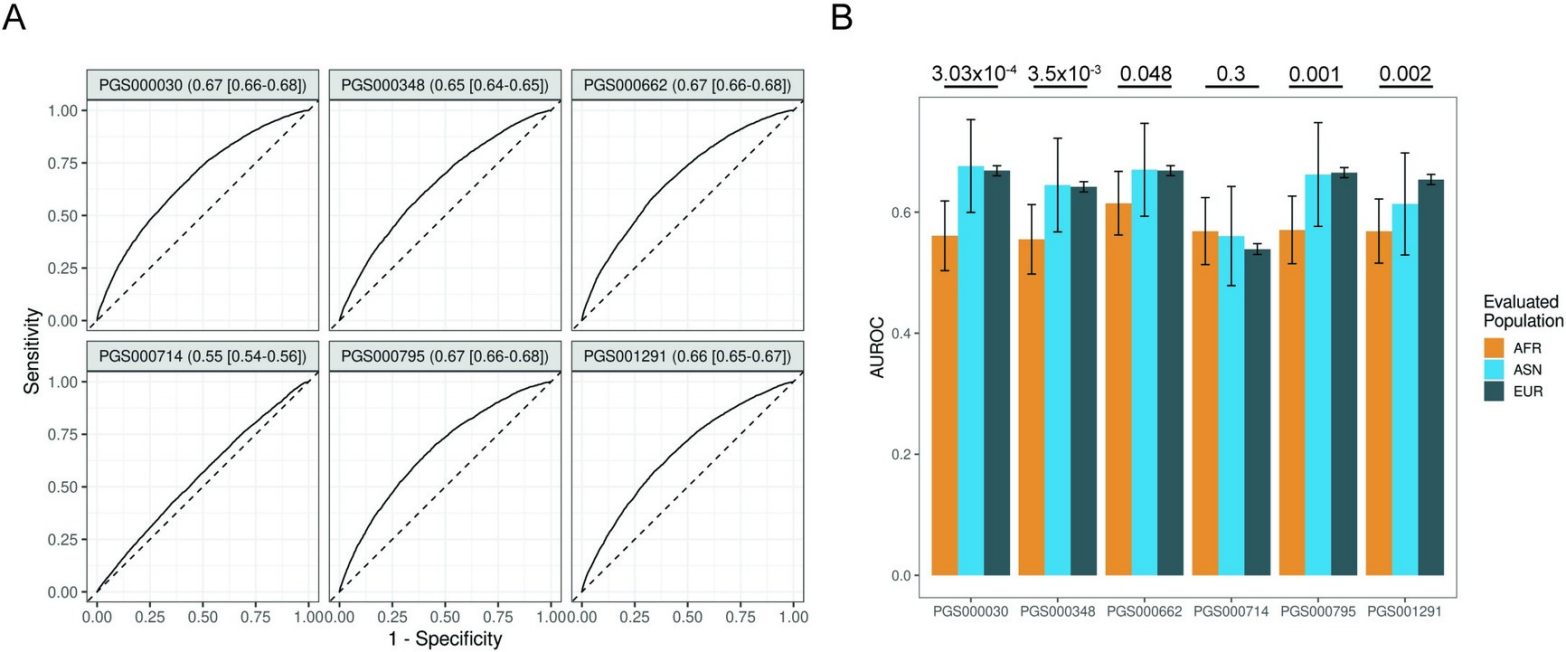
(A) Evaluation of published prostate cancer polygenic scores sourced from PGScatalog without stratifying by ancestry in the UK Biobank testing dataset. (B) Evaluation of published prostate cancer polygenic scores sourced from PGScatalog after stratifying by ancestry in the UK Biobank testing dataset. Confidence intervals were obtained by bootstrapping the AUC 2000 times. p values were obtained from De-Long’s test.

We found that prediction AUROCs ranged from 0.55–0.67 (**Fig 2A**) when evaluating the entire cohort and compared them to published results when available. We note that our evaluation resulted in comparable AUROCs to those reported in the original publications. Population-specific evaluation revealed the highest average AUROC for EUR predictions (0.63 [0.58–0.69], mean [95% CI]) across all evaluated PRSs. Interestingly, AUROC for ASN predictions was not significantly different from EUR (0.64 [0.59–0.68], p = 0.46). However, AFR participants had poorer AUROC than EUR (0.55 [0.52–0.58] vs 0.63 [0.58–0.69], p = 0.02, paired t test) (**S2A Fig**). Moreover, we find that all tested scores, apart from PGS000714, were associated with worse predictive power in AFR (**Fig 2B**, p < 0.05, DeLong’s test for receiver operator characteristic curves).

Despite these overall lower performances, we find that an ancestry-informed PRS (PGS000662) resulted in improved prediction in AFR participants (AUROC 0.59) relative to other PRSs. We estimated AFR-specific SNP weights by multiplying the SNP beta (as calculated by Conti et al. [10]) by the AFR allele frequency to better understand variants that may enhance performance in AFR individuals. This revealed that rs10090154, rs72725879, and rs11263763 were the top contributing SNPs for prediction in AFR. rs10090154 and rs72725879 are located on the 8q24 AFR risk locus near the *MYC* oncogene [11,20]. Fine mapping in a European cohort has identified the association between rs11263763, located on 17q12, and prostate cancer susceptibility [21]. Given that an ancestry-informed meta-analysis (PGS000662) resulted in improved prostate cancer risk prediction in AFR, we hypothesized that additional rigorous adjustment of cross-population linkage disequilibrium and population allele may improve predictive power in AFR men.

### Generation of polygenic risk scores

**Fig 3:**
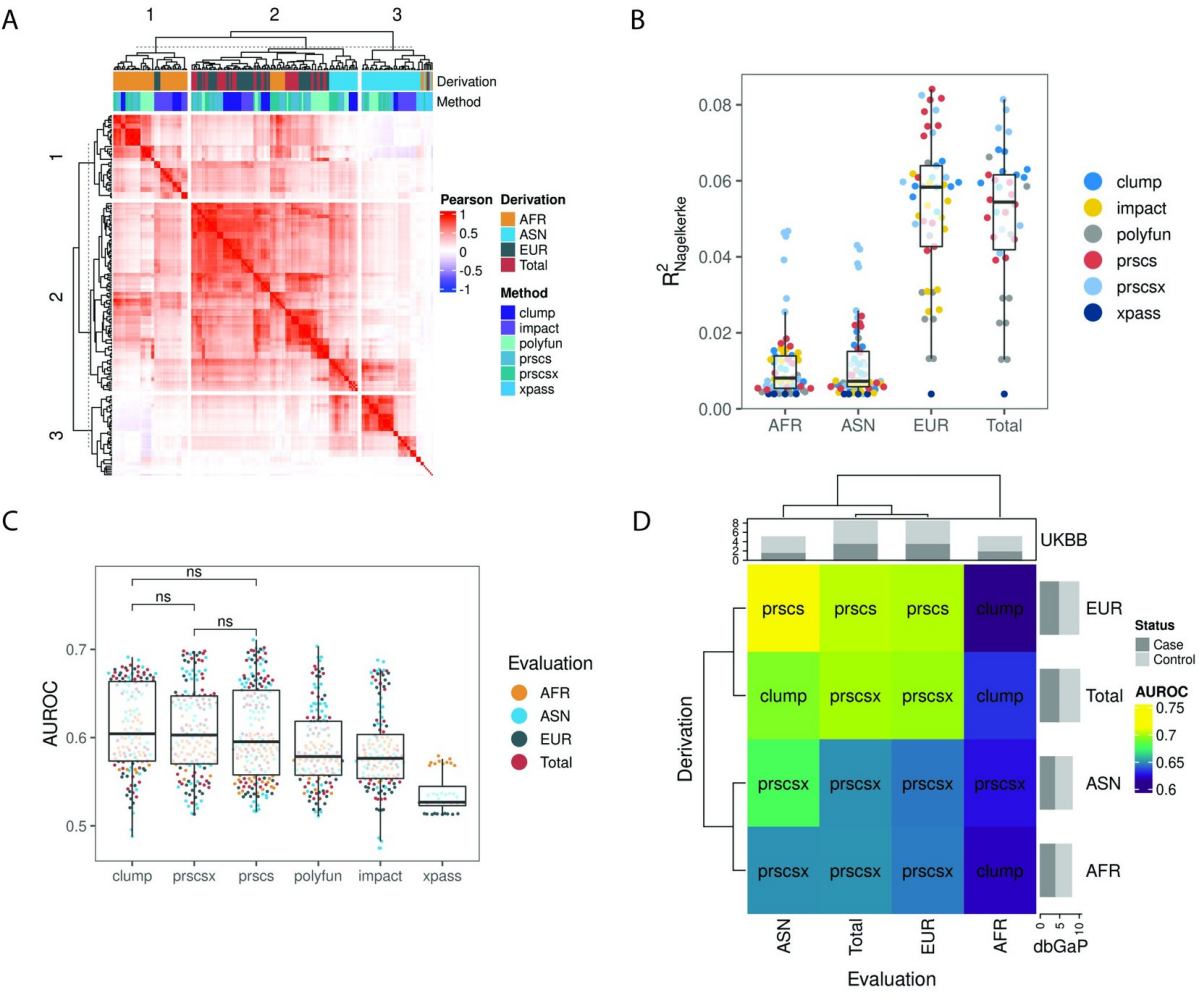
(A) Pairwise Pearson correlation matrix of 206 scores generated. (B) Evaluation of Nagelkerke’s pseudo R^2^ based on models trained on summary statistics adjusted for specific ancestries. (C) Evaluation of AUROC in the UK Biobank testing cohort. (D) Identification of polygenic scores associated with highest AUROC for each evaluation-derivation ancestry pair. Text within squares indicates associated algorithm. Top and right sample size annotations are in log scale.

As most existing PRSs were developed in majority EUR populations and were linked to suboptimal performance in African ancestry, we hypothesized that appropriately adjusting for corss-population linkage disequilibrium may improve disease prediction across populations. To develop such an improved PRS, we employed clumping, PRScs, PRScsx, IMPACT, PolyFun, and XPASS to generate scores across populations and tuning parameters (Methods). This process generated 206 unique scores that were designed for prediction in population specific (pairwise AFR, ASN, EUR) or agnostic contexts (Total). Indeed, we found that scores were positively correlated with each other (**Fig 3A**, mean Pearson *r* 0.266[0.263–0.27]). Although the scores were correlated, hierarchical clustering revealed population-specific clustering (*χ*^*2*^ p < 2.2x10^-16^) suggesting that the methods may not adequately adjust for ancestry.

Next, we similarly trained logistic models and evaluated model fit with Nagelkerke’s pseudo R^2^. Unsurprisingly, scores derived for EUR were associated with the highest variance explained (**Fig 3B and S4 Table**). We calculated a relative R^2^ metric to identify optimal methods for ancestry-adjusted scoring. This involved dividing all R^2^ values by the mean R^2^ associated with clumping in a population-specific manner. This revealed that in AFR and ASN, PRScsx was the only method that consistently explained greater variance than clumping alone (**S3A Fig**).

We repeated the model training and evaluation in propensity score matched data to mitigate the effect of any potential confounding from environmental factors. We found that it was not essential to match cases and controls, as the matched results largely aligned with the unmatched ones (**S3B Fig**). Our analysis utilized unmatched data to maximize statistical power and evaluated the prediction accuracy of all scores in the held-out dataset. Pairwise evaluation (all scores evaluated in all target populations) identified that clumping (0.612[0.603–0.62]), PRScsx (0.608[0.6–0.616]) and PRScs (0.605[0.598–0.613]) led to the highest AUROC (**Fig 3C and S4 Table**). Next, we identified scores that performed the best in a population specific manner (**Fig 3D**). Interestingly, the best performing scores did not correct for inter-population LD patterns and were associated with summary statistics from larger GWASs. We identified that clumping the combined summary statistics generated by Conti et al. [10], led to the highest AUROC when predicting disease in AFR (0.631[0.577–0.685]). In contrast, ASN were associated with the highest AUROC when PRScs was applied to EUR summary statistics (0.712[0.634–0.788]). While the difference between models that explained the greatest variance and those that had the highest AUROC was not statistically significant (**S3C Fig**, AFR p = 0.2, ASN p = 0.22), potentially due to sample size, our findings suggest that summary statistics from larger GWASs may be more informative than ancestry adjusted summary statistics from smaller ones.

### Integration of clinical risk factors

**Fig 4:**
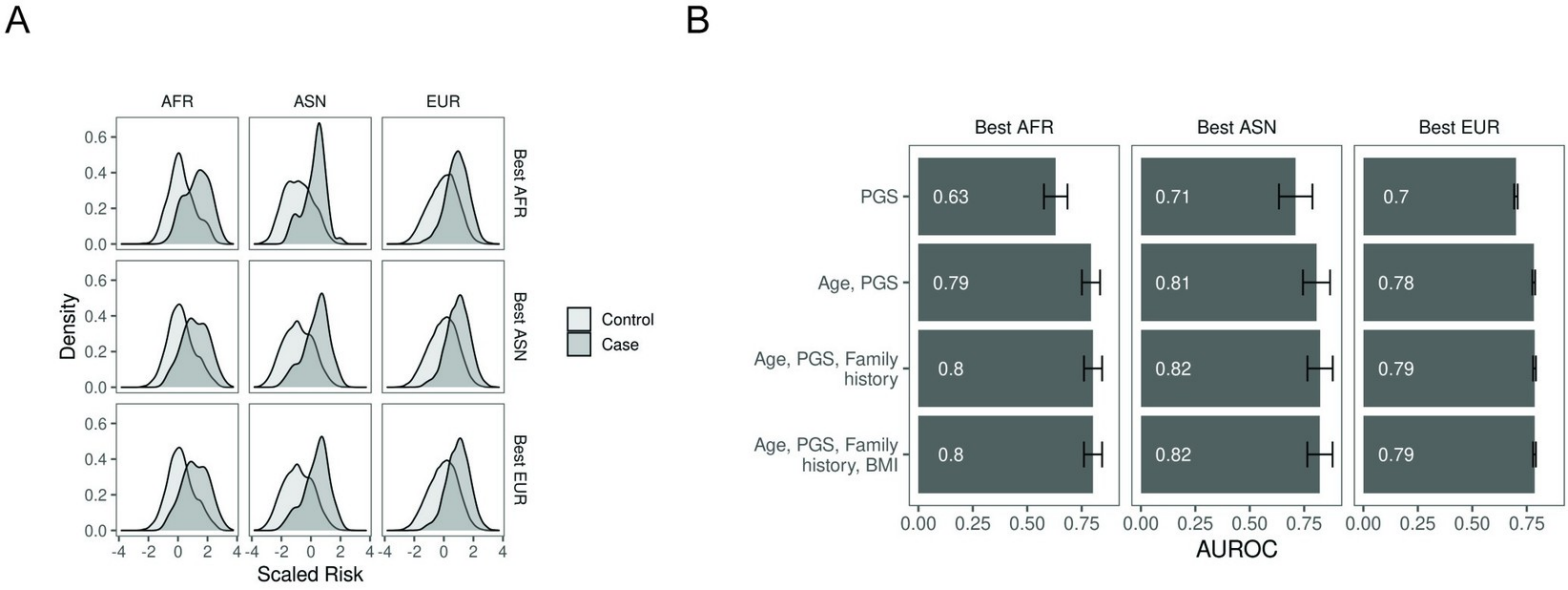
(A) Integrated risk score containing polygenic risk, age, family history and BMI for scores associated with highest population-specific performance. Best AFR: total-clumping; best EUR and best EAS: EUR-PRScs. (B) Predictive power associated with the inclusion of risk factors.

To evaluate the polygenic risk scores in models that reflect potential clinical utility, we fit a model that included top performing PRSs, age at assessment, PCa family history, and body mass index. These models, when evaluated in a population specific manner, drastically improved prostate cancer prediction (**Fig 4A**). Our analysis revealed that across all ancestries, the addition of age significantly increased the AUROC of a PRS-only model (**Fig 4B**, p < 0.05). The addition of additional risk factors did not improve predictive power substantially. Interestingly, the AUROC increase associated with the inclusion of age was the greatest in AFR (Δ AUROC = 0.16). It is possible that this increase in AUROC is due to unmatched cases and controls. However, we find that cases were older than controls across all populations (**S4A Fig**). Next, we explored whether aggregating all computed polygenic risk scores could improve disease classification using a deep learning framework. We found that employing a deep learning model resulted in performance similar to single-sourced polygenic risk scores (AUROC AFR = 0.79, EUR = 0.78, ASN = 0.81, Total = 0.79, **S4B Fig**). Taken together, we find that age and PRS alone are sufficient to predict prostate cancer at AUROCs greater than 0.75 across all ancestries.

### Age as a disease risk modifier

In exploring the role of age as a potential modifier of prostate cancer risk, we investigated its influence based on previous findings. Conti et al. (2021) [10] demonstrated that individuals younger than 55 face a heightened risk of disease compared to their older counterparts, especially among high-risk individuals. Our analysis employed logistic models that incorporated an interaction between polygenic risk scores and age categories (≤ 55 vs > 55). Although our study did not establish age as a significant modifier of disease risk among African and Asian populations, possibly due to the limitations in sample size, we observed that 78 out of 206 analyzed scores indicated an elevated risk in individuals under 55 years (Odds ratio > 1, FDR < 0.05, **S4C Fig**). Indeed, the models including the PRS-age interaction yielded a better fit compared to models without this interaction (Likelihood ratio test FDR < 0.05). Taken together, our findings suggest that age is a disease risk modifier in patients younger than 55 years.

### Validation in All of Us

**Fig 5:**
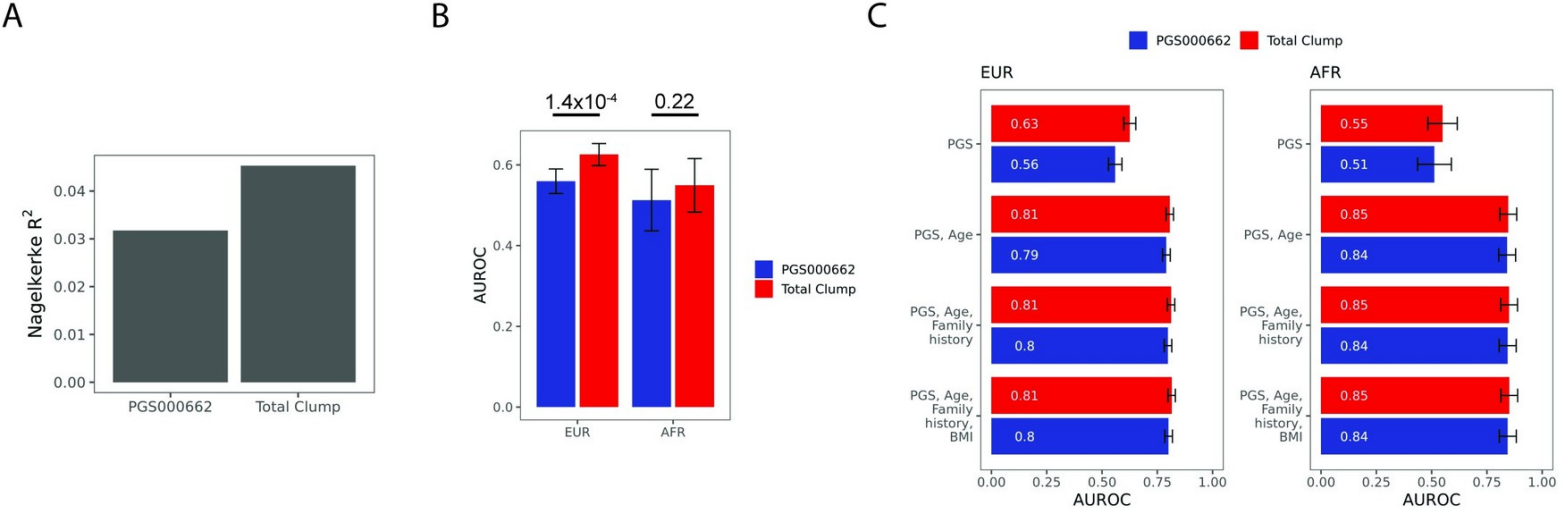
(A) Evaluation of Nagelkerke’s pseudo-R^2^ in models trained in the All of Us dataset. (B) Prediction AUROC of polygenic scores alone in the All of Us testing dataset. (C) Prediction AUROC of polygenic scores and age in the All of Us testing dataset.

To produce out-of-sample validation results, we assayed PRS performance of the top UK biobank score (clumping) in the All of Us (v6) dataset which is annotated with genetic ancestry (**S5A Fig**). This process involved array based genotyping data for 28,704 AFR and EUR participants. We could not reliably evaluate ASN individuals due the low sample size of cases. The dataset, while not imputed, contains measurements for 1,824,517 unique biallelic genotypes. We scored these genotypes with the UK Biobank PRS associated with the highest AUROC in AFR (Total clump) as well as the 269-SNP score from Conti et al. [10]. A random ancestry-and-case-stratified 2:1 split of these data yielded training and testing datasets. Fitting logistic models for these scores revealed that PGS000662 was associated with the poorer pseudo R^2^ (**Fig 5A**, 0.035 vs 0.045). Indeed, we found that clumping was associated with higher AUROC in AFR, although not statistically significant (**Fig 5B**, 0.55 vs 0.513, p = 0.22). Statistical significance was, however, achieved when evaluating EUR (0.63 vs 0.56, p = 1.4 x 10^−4^).

Next, we examined the effect of the addition of clinical risk factors in our models. We note that AFR cases and controls are younger than their EUR counterparts (**S5B Fig**). Predictive performance of trained models drastically improved upon integration of participant age and polygenic score. Similar to our findings in the UK Biobank cohort, the addition of family history and BMI did not increase predictive power (**Fig 5C**). Taken together, we demonstrate the utility of scores generated from the UK Biobank dataset and show that genetic risk coupled with participant age is sufficient to predict prostate cancer.

## Discussion

Recent advances in statistical genetics have led to improved methods for cross-ancestry PRS disease risk prediction. However, these improved models have not been formally assessed for their predictive performance in prostate cancer. In our comprehensive analysis across two biobank-scale datasets, we benchmarked six algorithms and find modest improvement over traditional clumping methods. These algorithms are based on variant prioritization, continuous shrinkage, joint analysis of summary statistics, functional annotation, fine mapping and the utilization of population-specific heritability estimates. To our knowledge, this is the first study to assess pan-ancestry polygenic risk for prostate cancer in the All of Us cohort.

In our benchmark, we find that hierarchical clustering is adequate to stratify polygenic scores by ancestry. These results suggest that the genetic correlation, measured by the correlation of polygenic scores, within individual ancestries is greater than that between prostate cancer cases. This finding may reflect the poor performance of ancestry-adjustment methods. Our study employed ancestry-correction using linkage disequilibrium maps from one of the largest publicly available sources [22]. It is possible that the sample sizes that derived these maps are inadequate, and that finer resolution mapping is required. Furthermore, inclusion of local ancestry haplotype models may improve predictive power of scores, especially for admixed individuals.

We find that while PRScsx explains the greatest amount of variance in logistic classifier models, it does not outperform other methods in classification accuracy on held-out data. While there is no consensus on PRS evaluation metrics and several studies report pseudo-R^2^, odds ratio or AUROC, we show that alternative metrics such as AUROC produce a different ranking of polygenic risk scores. Lastly, we note that polygenic score and the age of an individual are sufficient for acceptable classification of prostate cancer cases and controls across ancestries.

Our work, however, is constrained by certain limitations. First, we limit the analysis to genotyping arrays to maintain consistency and maximize sample size across the UK Biobank and All of Us cohorts. Genotype imputation has been performed for UK Biobank, but not for All of Us. Discrepancies in processing pipelines between derivation and validation data may limit the generalizability of our findings. Second, although we identified genetic ancestry, we could not perfectly account for population structure, admixture and subpopulations. For example, we grouped East Asian and South Asian individuals together to maximize the sample size of a population with low prostate cancer incidence. Additionally, it is possible that individuals annotated as AFR may represent distinct subpopulations, considering the vast genetic diversity within Africa, and the dissimilarity may be heightened between continents (Europe vs North America) [23]. Moreover, ancestry-bias correcting algorithms assume homogenous population structure for optimal performance. However, most individuals are admixed [24], and each admixture component has further fine-scale population structure that may prevent portability (*e*.*g*. Saharan vs Sub-Saharan ancestry). Third, although several ancestry-adjustment algorithms exist [25–28], we limited our study to six commonly used ones. These algorithms are complex and require fine-tuned parameterization for optimal implementation. Although we performed a robust benchmark and evaluated several parameters, a comprehensive sweep or parameters was not possible. However, based on our results, it seems unlikely that additional tuning would significantly alter prediction accuracy.

Taken together, our findings highlight the necessity for advanced computational strategies that more accurately reflect the genetic diversity across populations. Recent reports suggest that multi-ancestry PRS generating strategies are trait-specific and that out-of-the-box methods may not generalize [29]. Careful integration of several strategies such as thresholding genetic correlation between the source and target population and sample size thresholding may yield improved risk prediction. Moreover, the integration of non-linear models could complement the linear strategies we tested. For example, transfer learning and XGBoost result in more accurate predictions across a variety of traits [27,30]. Additionally, it is possible that whole genome sequencing, rather than array-based genotyping, may capture additional ancestry-biased causal variants, yielding improved risk prediction.

Given the dramatic diversity in incidence and outcome data across ancestry groups, poorly performing risk prediction tools may compromise risk stratification and patient care, particularly for those most likely to be afflicted. Our work highlights the need for additional algorithms to predict risk across populations as well as the sampling of diverse cohorts.

## Methods

### Data acquisition

The primary derivation source of data was the UK Biobank, a large, prospective, general-health study [31]. Approximately 500,000 individuals aged 40–69 were enrolled between the years 2006–2010. A diverse range of data was acquired during assessment, including electronic health records, diet and exercise habits, anthropometric measurements, self-reported disease history and blood samples [32]. Genotypic data for each individual was obtained by processing the blood sample with either the UK Biobank Axiom Array or UK BiLEVE Array. The resulting array data was imputed to 93,095,688 variants through the application of the IMPUTE2 program. We identified an individual as having prostate cancer if they either self-reported “prostate cancer” or their electronic health records contained an ICD-10 code C61 or an ICD-9 code 185. The electronic health records are continuously updated during inpatient hospitalizations and are available starting before each individual’s date of baseline assessment.

Genetic associations to prostate cancer were acquired from the ELLIPSE Prostate Cancer Meta-Analysis [10]. The data from this meta-analysis was deposited within dbGaP (phs001081.v1.p1). After approval (on Sep16, 2021) we downloaded the genome-wide association study (GWAS) summary statistics that were separately computed for four different ancestry-defined populations: European, African, Asian and Total. These four sets of summary statistics underwent standard quality control procedures which removed variants with missing statistics, duplicated rs IDs, alleles that were not A/C/T/G, ambiguous, not found in the UK Biobank imputed set, or that would not properly flip to match the alleles of the UK Biobank [33]. After rigorous quality control, all four sets of summary statistics contained more than five million variants.

The data source for score validation was the All of Us cohort (v6). Whole genome sequencing and genotype array data were available for 98,560 and 165,080 individuals respectively. We limited our study the genotype array data to maximize sample size and consistency with the UK Biobank. Genetic ancestry estimation and genotype quality control were performed by the All of Us research group prior to data acquisition. Cases were identified by medical history of prostate cancer while males without disease history were selected as controls. This yielded a total of 1,458 cases (213 African ancestry and 1245 European ancestry) and 27,246 controls (8,915 African ancestry and 18,331 European ancestry) with complete genotype data present. Prior to scoring the All of Us cohort, genetic scores were lifted over from the hg19 to hg38 genome reference.

### Summary statistic adjustment

The GWAS summary statistics were adjusted such that they could be used to calculate a maximally accurate polygenic risk score. We completed this adjustment with methods that were both population-aware, attempting to make the ultimate polygenic risk score portable to multiple populations, and population-unaware.

The two methods that were not population-aware include clumping and PRScs [15,34]. Clumping selects the variants with the lowest P-Value in a given linkage disequilibrium defined region and PRScs implements continuous shrinking about a window of variants in an attempt to replicate whole genome regression results. Both of these methods were applied to each of the four sets of summary statistics under multiple parameterizations as recommended by the software authors. For clumping, we included pairwise combinations p value thresholds of 1x10^-8^, 1x10^-6^, 1x10^-4^ and 1x10^-2^, and R^2^ thresholds of 0.1, 0.25 and 0.5. For PRScs, we tested phi values ranging from 1x10^-8^ to 1. Therefore, the PRScs method would lead to eight polygenic risk scores for each population and the clumping methods would lead to twelve.

Four methods that were population-aware include PRScsx, IMPACT, XPASS, and PolyFun [13,14,16,17]. PRScsx considered all the sets of summary statistics simultaneously (except for the set derived from the total population). By applying the same fundamental methodology as the previous PRScs method it would eventually create eight polygenic risk scores. The IMPACT method required the estimation of partial heritabilities combined with functional annotation. The functional annotation that corresponded to the greatest heritability would restrict variants that would take part in an otherwise standard clumping procedure. Using pairwise combinations of p value thresholds (1x10^-8^ and 1x10^-3^), R^2^ thresholds (0.1 and 0.5) and IMPACT annotation thresholds (0.9, 0.95 and 0.99), ultimately, 12 polygenic risk scores would be created for each of the four sets of summary statistics. However, two scores (one for AFR and one for ASN) could not be generated due to errors in the software. XPASS compared each of the four sets of summary statistics to either the Total or European set of summary statistics. By utilizing population specific heritability estimates XPASS can reportedly improve upon the standard clumping procedure and ultimately generate up to one polygenic risk score for each set of summary statistics. We used default parameterization was not required and this generated a single score per population. Lastly, PolyFun attempts to compare each of the five sets of summary statistics in a pairwise fashion to each of the other set of summary statistics. PolyFun attempts to apply fine-mapping principles to improve upon summary statistic adjustment. Multiple parameterizations created nine potential PolyFun associated polygenic risk score for each set of summary statistics.

Throughout the adjustment process, a collection of genotypes that represented the linkage disequilibrium patterns of a population were required. In these instances, we utilized a subset of the individuals from the UK Biobank with the corresponding population. Further information regarding the methodology and implementation of all the six adjustment methods are available through their respective publication. We attempted to faithfully replicate the intended use of each method and provide the exact code that was written in the process.

### Calculation of polygenic risk scores

After the adjustment of summary statistics, a total of 206 polygenic risk scores could potentially be created for each individual in the UK Biobank. We therefore calculated all 206 of these polygenic risk scores for all individuals, even though most of the polygenic risk scores were only designed for use in a specific population. Specifically, the PLINK utility’s “—score” command computed the polygenic risk score.

### Model fit and evaluation

Logistic models were with the glm function as implemented in caret using 5-fold cross validation. In all cases, we used “summaryFunction = twoClassSummary, metric = ROC”. Evaluation of model fit in the training dataset involved calculation of Nagelkerke’s R^2^ as implemented in “fmsb::NagelkerkeR2”. A receiver operator characteristics curve as implemented in “pROC::roc” was used to calculate the area under the curve. Specifically, we used “ci.auc” for estimating bootstrapped confidence intervals and “roc.test” to compare curves. When evaluated, sensitivity and specificity were identified at the Youden index.

### Deep learning model to aggregate polygenic risk scores

We trained a multilayer perceptron using PyTorch (v2.2.0) to test if aggregating polygenic risk scores would improve disease classification across ancestries. The architecture consisted of an input layer, two fully connected hidden layers (128 and 64 neurons, each with L1 regularization of 0.1) and a final dense layer with sigmoid activation. The model included dropout (0.1) and batch normalization between layers. Model training involved 5-fold cross validation and included randomly splitting the dataset into training, validation, and testing data. Optimization was performed using Adam with a learning rate of 0.0001, and the objective was to minimize binary cross-entropy loss. The model was trained for a maximum of 100 epochs, where each epoch was fit over a total of 1000 batches. An early stopping threshold of 10 epochs was set to prevent over-fitting based on validation loss, where the best model weights were restored upon early stopping. AUROC was computed using cross-validation predictions.

### Statistical analysis

Unless otherwise reported, Wilcoxon-Rank Sum test was used to compare groups and statistical significance was considered as p < 0.05.

## Supporting information

S1 Fig (A) Published or adjusted summary statistics were used to score the entire UK Biobank cohort. The cohort was subsequently split into training and testing datasets (2:1 ratio). Logistic models were fit on the training dataset using 10-fold cross validation (repeated 10 times) and evaluated in the testing data. Evaluations were conducted in an ancestry-agnostic and ancestry-aware manner. Additional validation was conducted in the All of Us cohort in a similar manner. (B) Ancestry-specific summary statistics (or total) were adjusted for all other ancestries in a pairwise manner. This resulted in 206 scores that were evaluated in all populations. All available ancestry-types of GWAS summary statistics (African, Asian, European, and total) were combined with six types of adjustment methods (Clump, prsCS, prsCSx, IMPACT, XPASS, PolyFun) and four types of ancestry-specific reference panels (AFR, EAS, EUR and total) to produce 206 sets of adjusted summary statistics. Each set of adjusted summary statistics were then combined with genotypic data for all males in the UK Biobank to generate polygenic risk scores
(TIF)

S2 Fig (A) Evaluation of ancestry associated AUROCs derived from PGScatalog summary statistics.
(TIF)

S3 Fig (A) Evaluation of Nagelkerke’s pseudo-R^2^ relative to the population-specific clumping mean. (B) Evaluation of case-control matching in the UK Biobank cohort. (C) ROC curves for polygenic scores associated with the highest AUROC and pseudo-R^2^.
(TIF)

S4 Fig (A) Age distribution in the UK biobank cohort. (B) Evaluation of model trained on all PRSs aggregated. (C) Evaluation of age as a disease risk modifier. Interaction between PRS and age buckets are shown. Points are colored based on the p value from a likelihood ratio test to compare the model with an interaction to the one without.
(TIF)

S5 Fig (A) Ancestry annotation in the All of Us dataset. (B) Age distribution in the All of Us dataset.
(TIF)

S1 Table Baseline characteristics of cases and controls in the UK biobank cohort. (XLSX)

S2 Table Characteristics of published polygenic risk scores. (XLSX)

S3 Table Baseline characteristics of training and testing data in the UK biobank cohort. (XLSX)

S4 Table Mean AUROC and Nagelkerke R^2^. (XLSX)
