# Supplementary figures and images for: Benchmarking multi-ancestry prostate cancer polygenic risk scores in a real-world cohort
Source: PLoS Comput Biol. 2024 Apr 10;20(4):e1011990. doi: 10.1371/journal.pcbi.1011990 (PMC11034641; doi:10.1371/journal.pcbi.1011990)

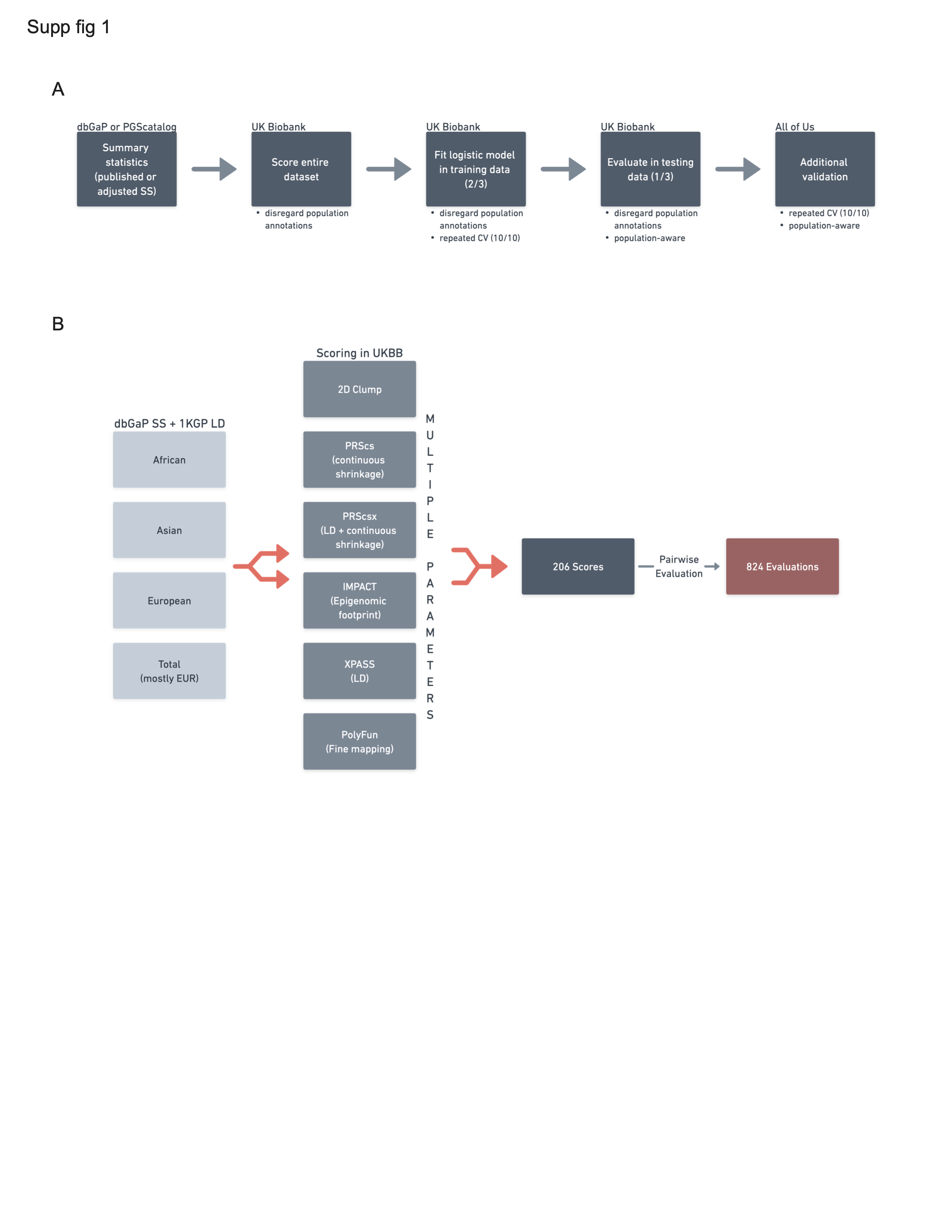

Supplement: S1 Fig — (A) Published or adjusted summary statistics were used to score the entire UK Biobank cohort. The cohort was subsequently split into training and testing datasets (2:1 ratio). Logistic models were fit on the training dataset using 10-fold cross validation (repeated 10 times) and evaluated in the testing data. Evaluations were conducted in an ancestry-agnostic and ancestry-aware manner. Additional validation was conducted in the All of Us cohort in a similar manner. (B) Ancestry-specific summary statistics (or total) were adjusted for all other ancestries in a pairwise manner. This resulted in 206 scores that were evaluated in all populations. All available ancestry-types of GWAS summary statistics (African, Asian, European, and total) were combined with six types of adjustment methods (Clump, prsCS, prsCSx, IMPACT, XPASS, PolyFun) and four types of ancestry-specific reference panels (AFR, EAS, EUR and total) to produce 206 sets of adjusted summary statistics. Each set of adjusted summary statistics were then combined with genotypic data for all males in the UK Biobank to generate polygenic risk scores (TIF) [file pcbi.1011990.s001.tif]

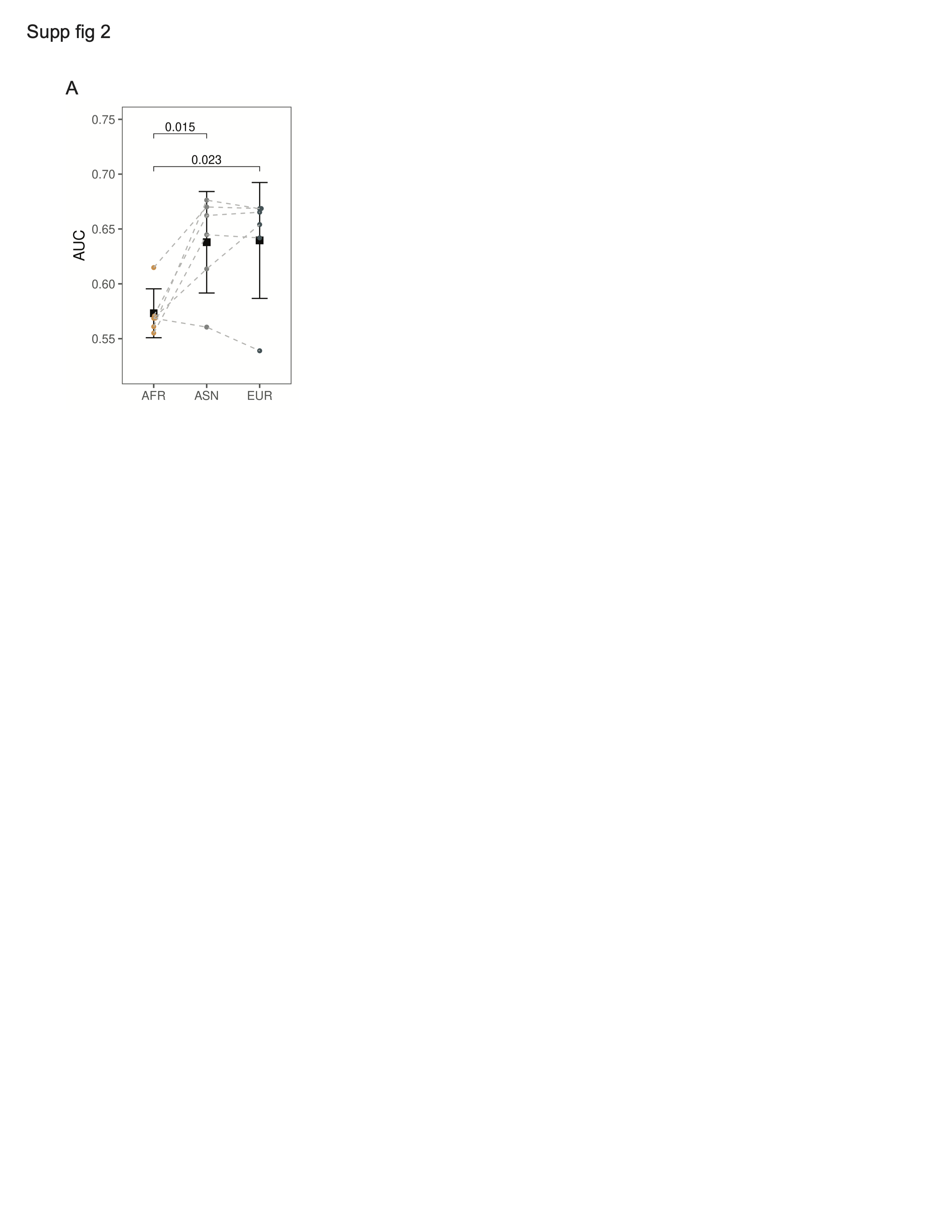

Supplement: S2 Fig — (A) Evaluation of ancestry associated AUROCs derived from PGScatalog summary statistics. (TIF) [file pcbi.1011990.s002.tif]

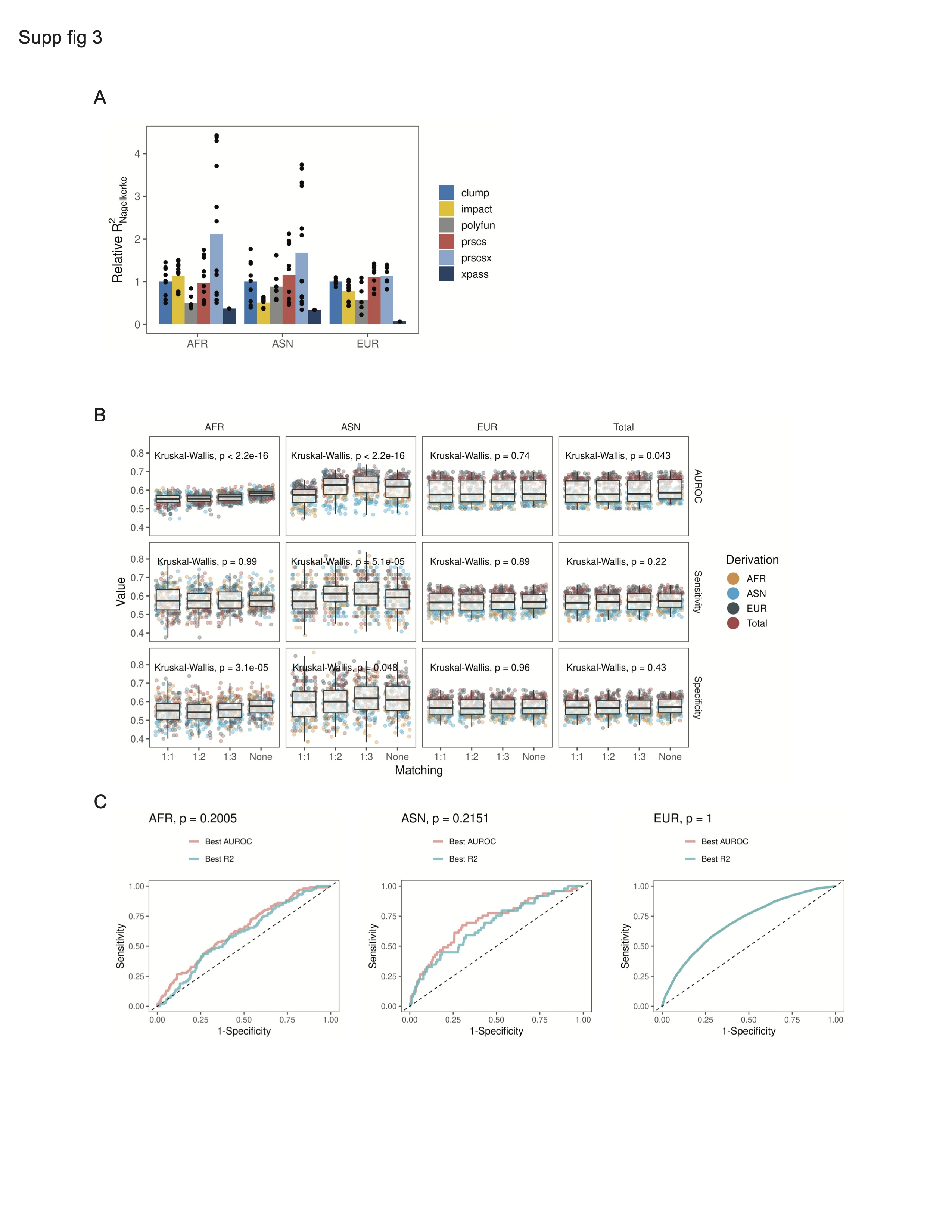

Supplement: S3 Fig — (A) Evaluation of Nagelkerke’s pseudo-R2 relative to the population-specific clumping mean. (B) Evaluation of case-control matching in the UK Biobank cohort. (C) ROC curves for polygenic scores associated with the highest AUROC and pseudo-R2. (TIF) [file pcbi.1011990.s003.tif]

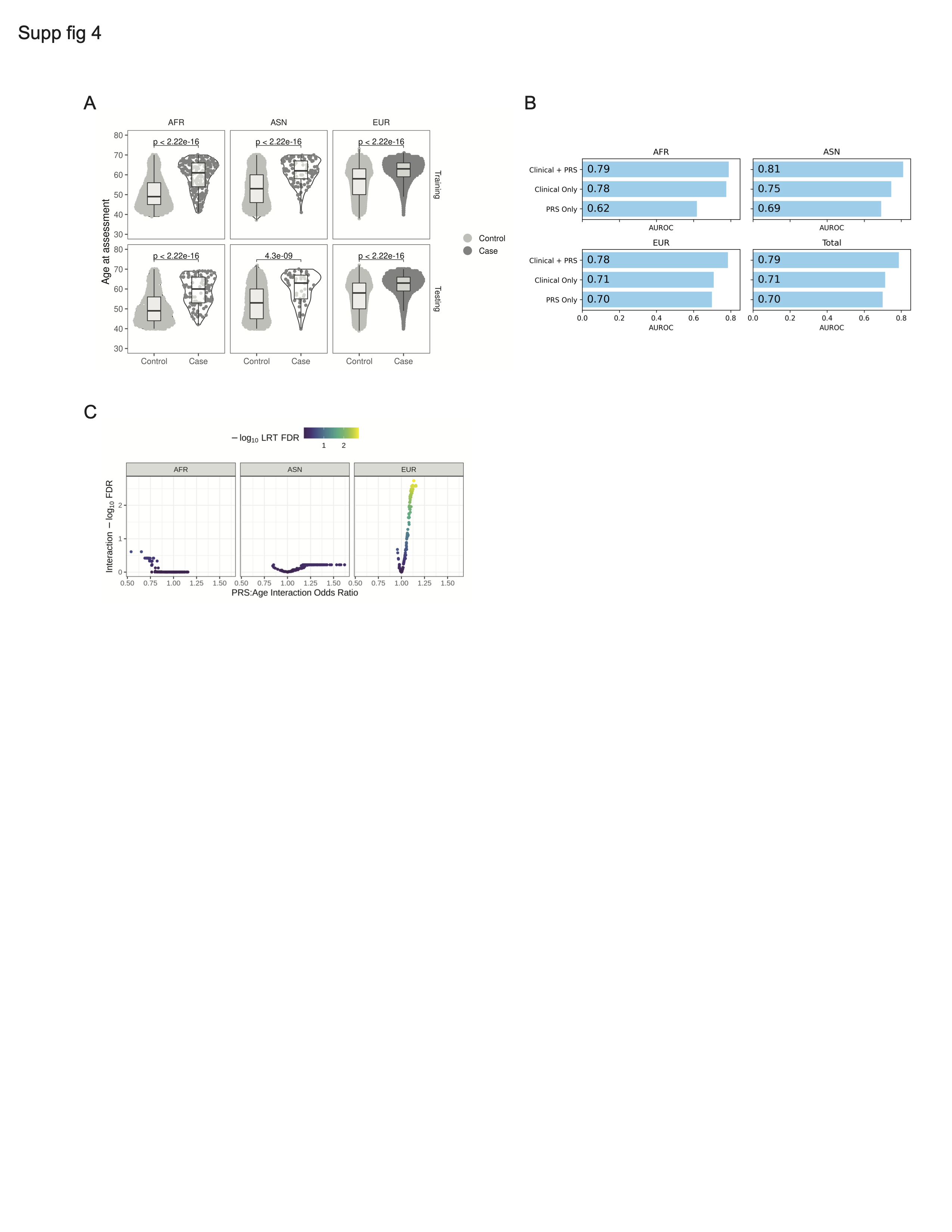

Supplement: S4 Fig — (A) Age distribution in the UK biobank cohort. (B) Evaluation of model trained on all PRSs aggregated. (C) Evaluation of age as a disease risk modifier. Interaction between PRS and age buckets are shown. Points are colored based on the p value from a likelihood ratio test to compare the model with an interaction to the one without. (TIF) [file pcbi.1011990.s004.tif]

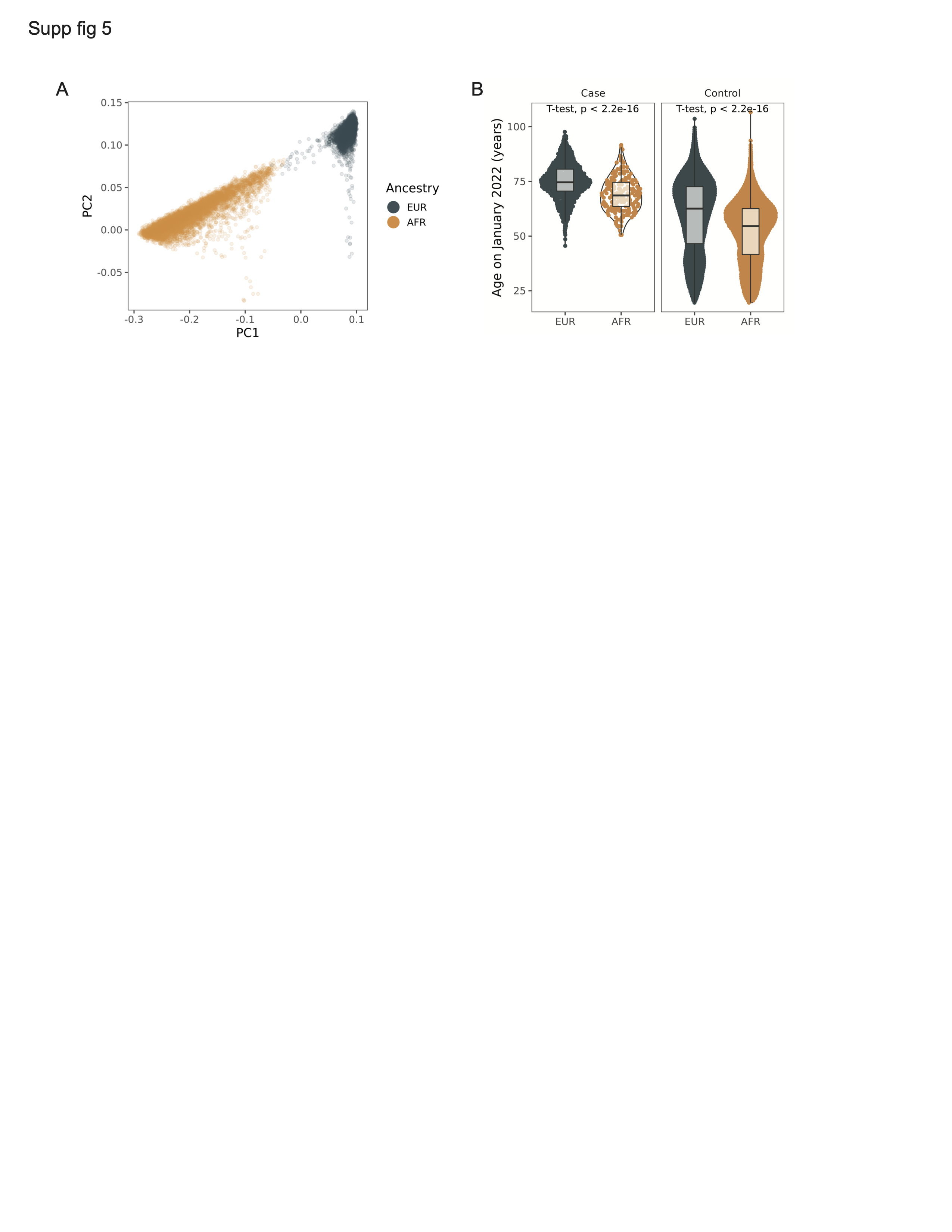

Supplement: S5 Fig — (A) Ancestry annotation in the All of Us dataset. (B) Age distribution in the All of Us dataset. (TIF) [file pcbi.1011990.s005.tif]
